# Harnessing Host-Vector Microbiome for Sustainable Plant Disease Management of Phloem-Limited Bacteria

**DOI:** 10.3389/fpls.2016.01423

**Published:** 2016-09-30

**Authors:** Pankaj Trivedi, Chanda Trivedi, Jasmine Grinyer, Ian C. Anderson, Brajesh K. Singh

**Affiliations:** ^1^Hawkesbury Institute for the Environment, Western Sydney University, Penrith SouthNSW, Australia; ^2^Global Centre for Land Based Innovation, Western Sydney University, Penrith SouthNSW, Australia

**Keywords:** pathogens, phytoplasma, *Candidatus* Liberibacter species, insects, biocontrol, microbial communities

## Abstract

Plant health and productivity is strongly influenced by their intimate interaction with deleterious and beneficial organisms, including microbes, and insects. Of the various plant diseases, insect-vectored diseases are of particular interest, including those caused by obligate parasites affecting plant phloem such as *Candidatus* (*Ca*.) Phytoplasma species and several species of *Ca.* Liberibacter. Recent studies on plant–microbe and plant–insect interactions of these pathogens have demonstrated that plant–microbe–insect interactions have far reaching consequences for the functioning and evolution of the organisms involved. These interactions take place within complex pathosystems and are shaped by a myriad of biotic and abiotic factors. However, our current understanding of these processes and their implications for the establishment and spread of insect-borne diseases remains limited. This article highlights the molecular, ecological, and evolutionary aspects of interactions among insects, plants, and their associated microbial communities with a focus on insect vectored and phloem-limited pathogens belonging to *Ca.* Phytoplasma and *Ca.* Liberibacter species. We propose that innovative and interdisciplinary research aimed at linking scales from the cellular to the community level will be vital for increasing our understanding of the mechanisms underpinning plant–insect–microbe interactions. Examination of such interactions could lead us to applied solutions for sustainable disease and pest management.

## Introduction

Plant pathogenic bacteria cause serious diseases for many major agriculture crops and fruit trees throughout the world (Vidhyasekaran, 2002), costing billions of dollars in damage annually (Pimentel et al., 2001). Of the various plant diseases, insect vectored diseases caused by obligate parasites of plant phloem are of particular interest (Bové and Garnier, 2003). These include the large and diverse group of *Candidatus* (*Ca*.) Phytoplasma species (transmitted by various hemipteran species including leafhoppers) and several species of *Ca.* Liberibacter (transmitted by the hemipteran species, psyllids). The fastidious nature of the members within *Ca.* Phytoplasma and *Ca.* Liberibacter hampers efforts to explore their epidemiology, the genetic mechanisms for disease manifestation, and for devising suitable control/prevention measures (Wang and Trivedi, 2013; Bertaccini et al., 2014). Infection by both groups of pathogens is often fatal, causing devastating damage to agricultural production around the world (Strauss, 2009; Munyaneza et al., 2010; Al-Sadi et al., 2012; Munyaneza, 2012; Wang and Trivedi, 2013). For example, phytoplasma epidemics among coconut palms have destroyed the livelihoods of many people in Africa and the Caribbean (Strauss, 2009). Huanglongbing (HLB) disease caused by *Ca.* Liberibacter spp. [including *Ca. L. asiaticus* (Las), *Ca. L. africanus*, and *Ca. L. americanus*) has had a devastating effect on the citrus industry worldwide (Wang and Trivedi, 2013). A relative, *Ca. L. solanacearem* causes zebra chip disease in potato, stunting and chlorosis in solanaceous species and foliage discoloration in carrots (Munyaneza et al., 2010; Munyaneza, 2012).

In recent years movement of propagative plant material and vegetable products has allowed the spread of both pest and pathogens around the world and their establishment in new areas where the conditions for disease development may be more favorable than in the area of origin (Wang and Trivedi, 2013). In addition, diseases transmitted by insects are expected to increase in frequency and spread to different localities due to global warming and climate change as future climates are advantageous to the cold-sensitive vectors (Hogenhout et al., 2008). Therefore, the development of robust and environmentally sustainable pest and pathogen control methods will become more important in the future.

**FIGURE 1:**
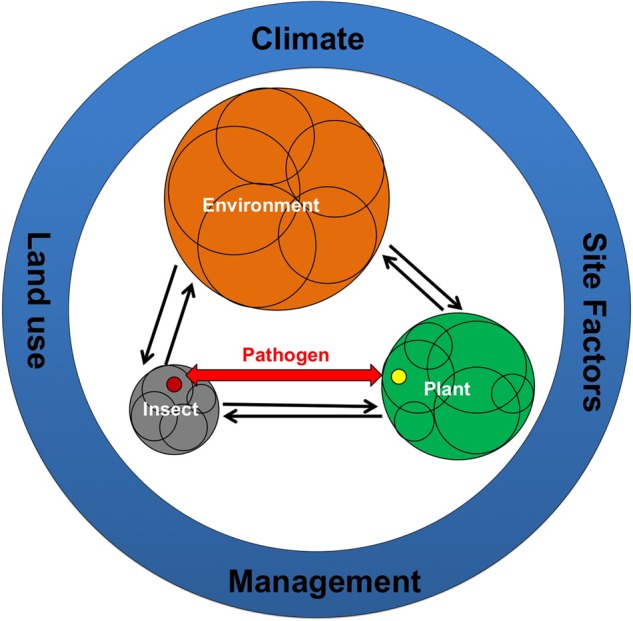
**Interactions between the environment, the hosts (insects and plants), their associated microbiome and obligate endophytic pathogens transmitted by insects (e.g., *Candidatus* (*Ca*.) Phytoplasma species and several species of *Ca.* Liberibacter).** Microbiome associated with hosts and environment is shown by different colored circles where the size of the circle represents greater numbers and diversity of the associated microbiome. Overlapping circles within hosts and environment represents different niches with specific microbiomes. Red colored arrow represents pathogen movement between plants and insects. Different color of pathogen in insect and plant is indicative of differential host adaptation strategies. Factors that influence host-environment-microbiome-pathogen interactions are shown in blue circles.

Much of our understanding of the molecular mechanism governing decisions between compatibility or defense in host–pathogen interactions come from the studies that incorporate “single species and monoculture”; typically reduced to one plant interacting with one experimentally added pathogen. This “reduced complexity” approach forms the basis of the “disease triangle” paradigm which conceptualizes the interaction between the host, pathogen, and environment by providing a framework used to explain disease causation factors (Francl, 2001). However, in nature microbes live in constant association with other microbial species, directly or indirectly interacting and creating multispecies communities (Sagaram et al., 2009; Bulgarelli et al., 2013; Turner et al., 2013; Chaparro et al., 2014). A paradigm shift that employs a broad community level view toward the evolution and ecology of plant pathogenic bacteria is now being considered. This view has the potential to provide new directions in disease control measures by unearthing the hidden ecology and pathogenic potential through a mechanistic understanding of pathogen interactions with their host and associated microbial communities (**Figure 1**).

Hosts (insect and plants) as well as the environment (such as soil) consist of complex and diverse microbiome that interacts with the respective micro-environments (**Figure 1**). The dynamic interaction of host and its associated microbiome together with environmental microbiota provides benefit to the host in terms of growth and fitness (Berg et al., 2014; Lebeis, 2014). Within the host and the environment, different niche habitats provide variable conditions for the development of specific microbiome (**Figure 1**). For example, microbial community differs between different sized aggregates in soil (Trivedi et al., 2015) and different tissues/parts in hosts (Edwards et al., 2015; Fonseca-García et al., 2016). Obligate endophytic pathogens that are vectored by insects reside in specific parts in their insects during various life stages (Douglas, 2015). In both plant and insect, pathogens interact with the microbiome of specific tissue and influence changes in host responses (Weiss and Aksoy, 2011). These host–environment–microbiome–pathogen interactions are influenced by climate, land use, management practices, and other environmental factors (soil properties, nutrient status, etc.; Mueller and Sachs, 2015). Selection of particular set of these environmental factors can affect the microbiome that will influence the outcome of pathogen infection. Similarly manipulation of host associated microbiome can lead to the development of novel disease management practices.

Although the unique features of phytoplasmas and *Ca.* Liberibacter spp. have long made them a subject of interest, the difficulty of *in vitro* culture has hindered their molecular characterization. In recent years the availability of genome sequences for several phytoplasma strains (Oshima et al., 2004; Bai et al., 2006; Kube et al., 2008; Tran-Nguyen et al., 2008), and *Ca.* Liberibacter spp. (Duan et al., 2009; Lin et al., 2011, 2015) have contributed significantly to our understanding of the biology of these pathogens. Analysis of these genomes has revealed that both groups have a very small genome (530–1350 kb for phytoplasma’s and 1190–1260 kb for *Ca.* Liberibacter spp.) and lack intact metabolic pathways involved in the biosynthesis of various fatty acids, sterols, amino acids, and nucleotides (Oshima et al., 2004; Tran-Nguyen et al., 2008; Duan et al., 2009; Wang and Trivedi, 2013). Consistent with their intracellular nature, phytoplasma and *Ca.* Liberibacter spp. lacks type III and type IV secretion systems (except for one type IVB system in some phytoplasma) as well as typical free-living or plant colonizing extracellular degradative enzymes. Although metabolic genes are scarce, the genomes of both groups of pathogens contain many genes encoding transporter systems, suggesting that these pathogens rely heavily upon nutrients and metabolites extracted from their host (Oshima et al., 2004; Bai et al., 2006; Kube et al., 2008; Tran-Nguyen et al., 2008; Duan et al., 2009; Wang and Trivedi, 2013). Considering the limited metabolic capacity, it is remarkable that they can interact with their hosts from two different kingdoms (Plantae and Animalia) and successfully colonize highly dissimilar environments. The consumption of metabolites by the pathogen greatly disturbs the metabolic balance of the host cell, causing disease symptoms. These altered conditions result in significant changes in the structure and function of stable multispecies communities associated with the host, wherein the augmented gene pool and the combined metabolic repertoire can influence pathogen survival and disease manifestation (Hosni et al., 2011).

This article explores the interactions of the phloem limited and insect vectored plant pathogens, Phytoplasma’s and *Ca.* Liberibacter spp. [mainly Liberibacter asiaticus (Las)] with their insect and plant host and their associated microbial community. We describe: (1) interactions of the insect associated microbial community with the pathogen(s); (2) differential gene expression that enables adaptation of pathogens to different hosts; (3) the modulation of host response by pathogens for their own transmission; (4) fluctuations in the structure and function of the plant associated microbiome in response to pathogen infection. We further highlight the potential for beneficial microbes within each plant and insect microbial community to be developed as biocontrol agents for the sustainable management of diseases caused by phytoplasma’s and *Ca.* Liberibacter spp.

## Insect-Associated Microbial Community and Its Interaction with Pathogens

The vascular tissues of plants are generally deficient in essential nutrients, therefore sap-feeding insects rely exclusively on their associations with bacterial symbionts to supplement their dietary needs (including amino acids, lipids, and vitamins; Buchner, 1965; Bourtzis and Miller, 2003). Studies have shown that sap feeding insects such as aphids and psyllids have significantly less microbial diversity as compared to xylophagous and leaf feeder insects (Ishii et al., 2013; Sugio et al., 2014). For these insects, most of the associated microbes are obligate or primary and facultative or secondary symbionts that are specifically associated with these different groups of sap feeders. The presence or absence of these bacteria could affect the competency of the insect vector to transmit pathogens or the life history traits of the insects themselves. For example obligate intracellular bacteria *Wolbachia* that are presumably found in up to 66% of all insects (Hilgenboecker et al., 2008) manipulate host reproduction by inducing cytoplasmic incompatibility, parthenogenesis, feminization, and male-killing (Stouthamer et al., 1999). Quantifying the presence of obligate endosymbionts and understanding the variety of facultative endosymbionts these insects utilize, may provide insights into the transmission of pathogens.

The *Ca.* Liberibacter asiaticus (Las) concentration within the insect was found to have a strong negative relationship with an endosymbiont residing in the syncytium of the mycetocyte (Fagen et al., 2012). Interestingly, the population of another bacteriocyte-associated bacteria, mycetocyte endosymbiont, was unaffected by Las acquisition. The variable effect of Las on endosymbiotic bacteria may be caused by its irregular distribution within the host causing certain bacteria to be displaced but not others (Fagen et al., 2012). Las titer had a positive relationship with the endosymbiotic community composed of *Wolbachia* which has been shown to alter host insect gene expression that creates a favorable intracellular environment for its growth (Hussain et al., 2011). A comparable mechanism may lead to increased *Wolbachia* and related increases in Las populations within its vector Asian citrus psyllid (ACP). Finding an increase in *Wolbachia* titer with Las infection indicates a more complicated mechanism than simple replacement of indigenous endosymbionts by Las. Ishii et al. (2013) reported strikingly complex endosymbiotic microbiota of the *Macrosteles* leafhoppers that vectored two genetically distinct phytoplasma’s. The microbiome of these leafhoppers included two obligate endosymbionts, “*Ca. Sulcia muelleri*” and “*Ca. Nasuia deltocephalinicola*,” and five facultative endosymbionts, *Wolbachia*, *Rickettsia*, *Burkholderia*, *Diplorickettsia*, and a novel bacterium belonging to the *Rickettsiaceae*. The highly complex endosymbiotic microbiota suggested ecological interactions between the obligate endosymbionts, the facultative endosymbionts, and the pathogenic phytoplasma’s within the same host insects that may affect the competence of insect vector. The role of insect-associated microbes in altering the transfer rate of pathogens has not yet been reported (Sugio et al., 2014). Filling this knowledge gap is key to understanding disease epidemiology and for improving disease control strategies.

## Pathogens Modulate Gene Expression During Transmission in Different Hosts

In order to proliferate and cause disease, insect vectored pathogens have to switch between the diverse environments of plants and insects (Chatterjee et al., 2008; Yan et al., 2013). These different environments have a dramatic effect on bacterial gene expression; specific genes whose products assist in survival are activated, whereas non-essential gene products in a particular environment are deactivated (Chowdhury et al., 1996). It has been suggested that virulence factors are expressed at different stages of the infection process and are dictated by the changing microenvironment of the host (Chowdhury et al., 1996).

The number of Las genes up-regulated in plants was higher when compared to the insect vector (Yan et al., 2013), while an opposite trend was observed for phytoplasma (Oshima et al., 2011; Makarova et al., 2015). One possible reason for this difference is the co-evolution of the pathogen, plant host and insect vector. *Ca.* Liberibacter spp. evolved from an ancestor in the plant-associated Rhizobiaceae family whereas phytoplasma’s are closely related to an animal-associated *Mycoplasma* or *Acholeplasma* spp. (Oshima et al., 2013). It can be proposed that *Ca.* Liberibacter spp. and Phytoplasma’s would have undergone adaptive, diversifying, and reductive evolutionary processes that would have made them more suitable for their interactions with specific plants and insects, respectively. The intimate association of *Ca.* Liberibacter spp. with plants as endophytes predisposes them to frequent encounters with herbivorous insects, providing ample opportunities to evolve alternative associations with insects (Nadarasah and Stavrinides, 2011). Similarly, associations between phytoplasma and insects provide opportunities for alternate associations with different plant species.

The expression levels of several transporter genes were differentially expressed between hosts in Aster Yellows phytoplasma witches’ broom (AY-WB) and *Ca. Phytoplasma asteris* OY-M strain of phytoplasma and Las. Zinc transporter genes were upregulated in insects for both the phytoplasma species, whereas for Las they were highly expressed in plants (Oshima et al., 2011; Yan et al., 2013; Makarova et al., 2015). For both groups of pathogens, multidrug efflux pumps were upregulated in plants (Oshima et al., 2011; Yan et al., 2013; Makarova et al., 2015) demonstrating host-specific genetic expression in order to adapt between two hosts.

After inoculation into the plant phloem by the insect, the pathogen encounters a change in osmolarity and must protect itself from dehydration and loss of turgor. Phytoplasma and Las deal with the problem of osmolarity through different mechanisms. For both pathogens, the genes for dealing with osmolarity were up-regulated in plants when compared to insects (Oshima et al., 2011; Yan et al., 2013; Makarova et al., 2015). The Las gene *proX*, involved in the transport of the most common osmoprotectants glycine betaine, was up-regulated in plants compared to their insect vectors psyllids (Yan et al., 2013). In AY-WB and *Ca. Phytoplasma asteris* OY-M the MscL channel that senses mechanical stretching of the membrane was significantly up-regulated when compared to the insect vector (Oshima et al., 2011; Makarova et al., 2015).

Analyses of the phytoplasma and *Ca.* Liberibacter spp. genomes have identified glucanase, serralysins, and hemolysin-like proteins as possible virulence factors (Bai et al., 2006; Duan et al., 2009; Wang and Trivedi, 2013). Hemolysins are bacterial toxins that act to form a transmembrane channel within the membrane of susceptible cells causing the leakage of ions, water, and low molecular weight molecules into the host cell (Gouaux, 1998). The expression of putative hemolysin genes were upregulated in the insects in AY-WB phytoplasma while in Las these genes were upregulated in plants (Yan et al., 2013; Makarova et al., 2015). Although no differential expression of this gene was found in another phytoplasma OY-M, it has been suggested that hemolysin may be involved in virulence and insect transmission in other insect transmitted plant pathogens (Wang et al., 2012). In Las, genes encoding a secreted metalloprotease serralysin were highly expressed in plants compared to the insect vector (Yan et al., 2013). Serralysin is postulated to aid bacterial survival in plants by modifying plant defense and nutrient uptake. Interestingly, many candidate secreted effectors proteins of Las postulated to modulate cellular functions for disease progression (Pitino et al., 2016) were upregulated in plants (Yan et al., 2013).

In Las, genes that encode enzymes involved in glycolysis were up-regulated in plants (Yan et al., 2013). The high expression of glycolysis-associated genes in plants indicates that Las can use glucose acquired from the host plant to generate energy for intracellular growth. In contrast, phytoplasma glycolytic genes were not differentially expressed in both hosts (Oshima et al., 2011; Makarova et al., 2015). In both AY-WB and OY-M strains, genes relevant to the malate and pyruvate pathways were markedly up-regulated in insects. Malate can be utilized as a sole source of energy by phytoplasma (Kube et al., 2012) and might serve as an important source of energy for phytoplasma’s when colonizing the leafhopper vector (Makarova et al., 2015).

Overall the studies on the gene expression profiles of phytoplasma and Las in plants and insects have reported a dramatic response to the diverse host environments when compared with environmental changes in other bacteria (Oshima et al., 2011). In general, responses were markedly different for Las and phytoplasma suggesting species and host-specific adaptations to the distinct environment of plant and insects. However, our understanding of the exact roles of these genes in host switching remain largely unknown. A clear understanding of the molecular basis of host switching can unlock the possibility for the development of novel methods in pest control for insect transmissible pathogen diseases.

## Pathogens Alter Plant Physiology and Morphology to Attract Vectors

It is common for the insect vectored pathogens to manipulate plant–insect interactions to enhance their own dissemination via effects on: (a) the quality of the primary host as a resource for the vector (Mauck et al., 2010), or; (b) the production of host-derived cues that mediate vector attraction (Lefèvre et al., 2006). Infection and subsequent disease manifestation in plants change the plant architecture and/or physiology that enhance both vector recruitment to infected plants and subsequent dispersal of the pathogen to healthy plants.

Effector proteins secreted by different species of phytoplasma induced the production of many leaves and stems in their hosts, creating a characteristic bushy appearance and converting plants into more attractive hosts for egg-laying and reproduction of leafhopper vectors (Hoshi et al., 2009; MacLean et al., 2011; Sugio et al., 2011). For example phytoplasma AY-WB produces a novel effector SAP54 that degrades the highly conserved transcription factors of the MADS-box family involved in flower development leading to the generation of sterile plants (MacLean et al., 2014). These sterile plants, which form leaves rather than flowers, are more attractive to leafhoppers. Similarly another virulence effector from OY-M, tengu-su inducer (TENGU) disrupts the auxin signaling pathway and induces dwarfism and witches’ broom symptoms that attract more insects (Hoshi et al., 2009; Sugio et al., 2011; Minato et al., 2014). The characteristic yellowing symptoms in Liberibacter and phytoplasma infected plants (Wang and Trivedi, 2013; Bertaccini et al., 2014) can also play a role in insect vector attraction.

Pathogens are also known to induce plant responses that modify behavior of the insect vector by altering the olfactory cues through changes in volatile and non-volatile secondary metabolites that insects use to locate suitable host plants (Orlovskis et al., 2015). For example, using a multitrophic system consisting of a phytoplasma [*Ca. Phytoplasma mali* (*Ca. P. mali*)], a host tree (*Malus domestica*), and a phloem-feeding insect (*Cacopsylla picta*), Mayer et al., 2008a,b) showed that infected apple plants released higher amounts of the sesquiterpene β-caryophyllene than uninfected plants. Newly hatched adults of *C. picta* were attracted by the odor of infected apple trees. Las infected plants produce significantly more methyl salicylate and less methyl anthranilate and D-limonene as compared to non-infected plants (Mann et al., 2012). Methyl salicylate was attractive to psyllids, while methyl anthranilate did not affect their behavior suggesting that odorants mediate psyllid preference. This apparent pathogen-mediated manipulation of vector behavior may facilitate pathogen spread. Feeding on citrus by ACP adults also induced release of methyl salicylate, suggesting that it may be a cue to reveal the location of conspecifics on host plants (Mann et al., 2012). Similar processes have been documented in other complex patho-systems (Shapiro et al., 2012) suggesting that detailed insights on the mechanisms driving such effects will have far-reaching implications both for basic ecology and for the management of disease processes in natural and agricultural settings. Further characterization of the infochemicals that are induced by plant pathogens to attract the vectors will lead to the development of new traps (such as sticky traps) for monitoring or even mass trapping of vectors for pest control.

## Impact of Pathogens on Plant Associated Microbial Communities

**FIGURE 2:**
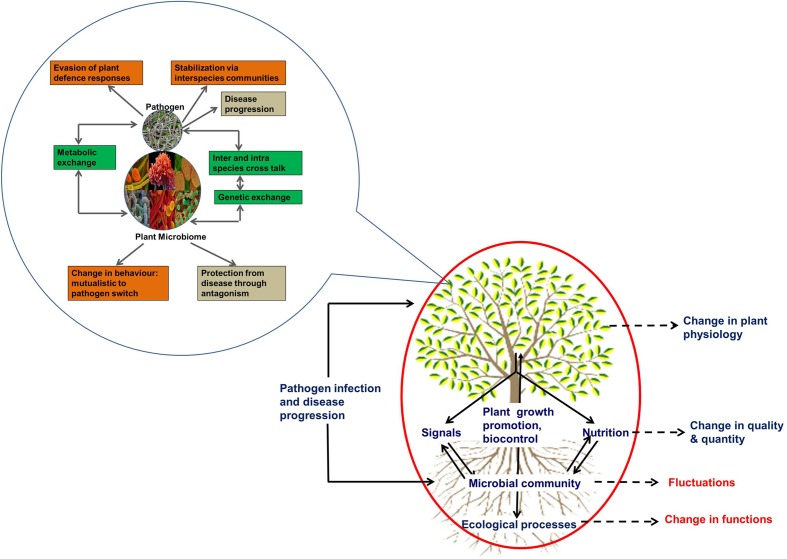
**Conceptual framework of the effect of plant disease.** The processes mediated by plant associated microbial communities are enclosed in the red circle. Black dotted arrows represent changes in various processes in response to pathogen infection and disease progression. Those shown in red color are poorly understood. The inset enclosed in blue circle shows the possible mechanisms involved in the interaction between incoming pathogens and resident microbes. The interactions are shown in green boxes. The changes in phenotypes are shown in orange boxes and the effects on the host are shown in black boxes.

Plants are associated with an astounding number and variety of microbes that interact with their hosts with different degrees of dependencies including competition, commensalism, mutualism, and parasitism (Garbeva et al., 2004; Bulgarelli et al., 2013; Philippot et al., 2013). It has been postulated that the disruption of multi-trophic plant–microbe–environment interactions under the influence of invading pathogen(s) will cause community reorganization and changes in local feedback interactions (**Figure 2**). However, there is a paucity of synthesized knowledge on the extent to which such community shifts may occur, the dynamics of these changes and the putative effects regarding the microbial mediated ecological functions (Trivedi et al., 2010, 2012). As the diversity and stability of plant-associated microbial communities heavily influence soil quality, plant production, and ecosystem processes, fluctuations in microbial community structure could have serious implications in ecosystem sustainability (**Figure 2**).

The structure of plant associated bacterial community changes in response to a variety of processes, and these shifts have been suggested to impact various ecosystem processes (e.g., nutrient recycling, decomposition) and/or the outcome of host–pathogen interactions (e.g., growth of pathogens, release of plant growth promoting rhizobacteria; Emmert and Handelsman, 1999; Trivedi et al., 2011; Philippot et al., 2013). Also, the interactions between plant-associated microbial communities and pathogens are not well understood, and our knowledge of the intimacy and decisiveness of such associations with respect to the behavior and survival of participating organisms is still in its infancy (Trivedi et al., 2010, 2012).

In general, pathogen triggers a cascade of reactions in plants, leading to the synthesis of defensive compounds which in turn enable it to withstand pathogen attack either directly (e.g., by structural or physiological modifications) or by mediating different plant signaling pathways (Lichtenthaler, 1998). The altered conditions after the pathogen attack could have variable effects on the survival and proliferation of different groups of plant-associated microbes. For example, infection by Las (Sagaram et al., 2009; Trivedi et al., 2010) and phytoplasma (Bulgari et al., 2011, 2014) has been reported to restructure the endophytic microbial community of their respective hosts. Pathogen infection caused a decrease in the overall bacterial diversity in the infected host (Trivedi et al., 2010; Bulgari et al., 2011). Interestingly, the abundance of bacteria belonging to *Sphingobacterium* was increased in the plants infected from Las and Phytoplasama (Trivedi et al., 2010; Bulgari et al., 2011). In general, it appears that infection by plant pathogens restructures the microbial community; many species show reduced levels, are not detected or are replaced by other indigenous populations, which can better tolerate/adapt to the stress condition and interact closely with the pathogen.

Interestingly obligate endophytic pathogens have been reported to restructure the native microbial community even when a direct competition effect is lacking. For example, significant changes in the microbial community structure of rhizosphere soil samples were observed in Las infected citrus (Trivedi et al., 2012). In this case, alteration in plant physiology leading to quantitative and qualitative changes in partitioning the photo-assimilates was the primary cause of the shift in microbial diversity of the diseased host. Typical rhizosphere inhibiting bacteria such as those belonging to Proteobacteria were significantly reduced in the infected plants suggesting that rhizosphere bacteria react more strongly to changes in plant physiology and exudation induced by pathogen infection.

In recent years, several reports have demonstrated profound shifts in the structure and composition of plant-associated microbial communities (Araújo et al., 2002; Reiter et al., 2002; Trivedi et al., 2010, 2011, 2012; Bulgari et al., 2011) in response to pathogen infection, however, the implication of these shifts on ecosystem functions are not well understood. Using Las and citrus huanglongbing as a model for pathogen–disease interactions that involve the blockage of vascular tissues, Trivedi et al. (2012) have reported that the introduction of pathogens into natural ecosystems perturbs the stability of the microbial community, thus affecting biogeochemical cycles that regulate soil fertility and ecosystem functions. Using comprehensive functional micro-array “GeoChip 4.0” authors showed that HLB disease has significant reduced abundance of functional guilds involved in key processes involved in nutrient cycling such as nitrogen cycling, carbon fixation, phosphorus utilization, metal homeostasis, and resistance. As the diversity and stability of the plant-associated microbial communities heavily influence soil and plant quality and ecosystem processes (Nannipieri et al., 2003; Garbeva et al., 2004), erosion of microbial diversity could have serious implications on the agro-ecosystem sustainability. In addition shrinking genetic and functional diversity in response to pathogen infection, will compromise the capacity of adaptive responses to further perturbation. These results pointed toward the beyond yield effect of plant diseases on ecosystem processes and suggested that in the long term, these fluctuations might have important implications for the productivity and sustainability of agro-ecosystems.

## Exploiting Host–Microbe–Pathogen Interactions for Disease Management

### The Potential of Insect Associated Microbiome for Pest Management

Currently, the management of diseases caused by phytoplasma and *Ca.* Liberibacter species is commonly based on the control of the insects, i.e., by spraying various insecticides, and on practices where the removal of symptomatic plants is undertaken (Tiwari et al., 2011; Wang and Trivedi, 2013). It is well recognized that the use of chemical insecticides as the main control strategy is not sustainable, and is known to have negative side-effects, including both environmental and biological effects (Qureshi and Stansly, 2007; Tiwari et al., 2011; Orduño-Cruz et al., 2015). Based on information on the insect associated microbial community Crotti et al. (2012) have proposed a “Microbial Resource Management (MRM)” that foresees the proper management of the microbial resource present in a given ecosystem in order to solve practical problems through the use of microorganisms. Some first steps of MRM applications have been already carried out on insect vectors, with the aim of defining the microbial community composition and functionality within the insects (Marzorati et al., 2006; Miller et al., 2006; Crotti et al., 2012). Researchers have reported various potential biological control bacteria associated with insect vectors that can provide opportunities for controlling these economically important vectors, either through potential paratransgenesis or cytoplasmic incompatibility (Powell and Tabachnick, 2014). The final aim is to propose a biocontrol approach based on the management of the microbial symbiont associated with the vector in order to counteract directly the pathogen or to reduce the vector competence.

Efforts are underway to develop mycoinsecticides for the biocontrol of ACP by the use of single-spore high-virulence strains of endophytic fungi *Isaria javanica* (Ayala-Zermeño et al., 2015; Gallou et al., 2016). This species has been reported to be associated with ACP and has been described as a pathogen of Lepidoptera, Coleoptera (Samson, 1974; Shimazu and Takatsuka, 2010) and the greenhouse whitefly of the order Hemiptera (Scorsetti et al., 2008). Application of conidial based formulations of endophytic fungi *Metarhizium anisopliae*, *Isaria fumosorosea* and *Hirstuella citriformis* resulted in high mortality of vectors of *Ca.* Liberibacter spp. such as ACP or *Bactericera cockerelli* (Tamayo-Mejía et al., 2014; Orduño-Cruz et al., 2015). Although the speed of kill caused by an entomopathogenic fungus is not comparable with that of a chemical insecticide, entomopathogenic fungi are known to reduce the feeding activity of infected hosts (Avery et al., 2009), resulting in reduced pathogen transfer, but this needs further experimental confirmation (Orduño-Cruz et al., 2015). Further research is required before the true potential of controlling insect vectors by biocontrol agents can be realized. Given the efficacy of biocontrol agents is reported to influenced by a range of parameters such as type of formulations, time and mode of applications and environmental and climate conditions, developing whole microbiome approach can potentially provide better disease control. However, this would require the development of effective tools to manipulate microbiome of the vector.

### The Potential of Plant Associated Microbiome for Increasing Plant Performance and Disease Resistance

**FIGURE 3:**
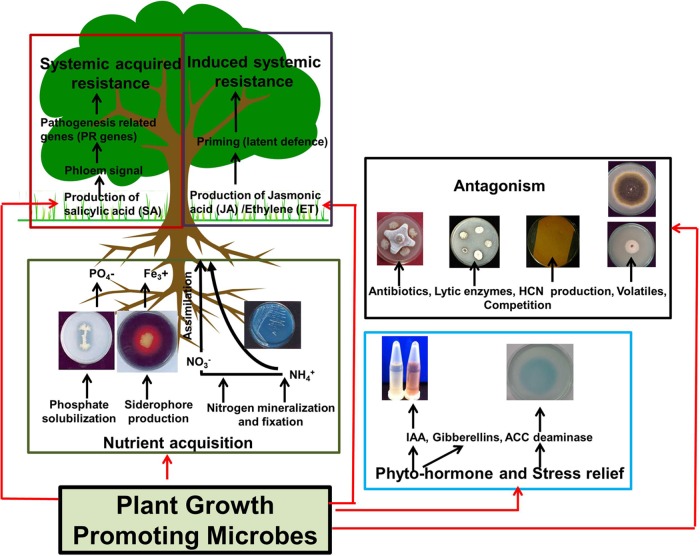
**Mechanisms used by plant growth promoting microbes (PGPM).** Different colored boxes represent individual mechanism by which PGPM’s can influence plant growth directly (i.e., nutrient acquisition and production of phyto-hormones or stress relieving enzymes) or indirectly (i.e, through direct antagonism or through the development of systemic acquired/induced systemic resistance). Note that these mechanisms represent general beneficial activities or PGPM’s and are not specific to *Ca.* Liberibacter spp. or phytoplasma infection. Details of the assays shown to demonstrate beneficial activities are provided in the supplementary section.

Plant-associated microbes which improve the fertility status of soil and contribute in augmenting overall plant growth and health known as Plant Growth Promoting Microbes (PGPM) are receiving increased attention for use as microbial inoculants in agriculture (Estrada-De Los Santos et al., 2001; Choudhary and Johri, 2009; Lugtenberg and Kamilova, 2009; Trivedi et al., 2011) (**Figure 3**). These microbes support plant health and growth by various mechanisms that include nutrient solubilisation and fixation, production of plant hormones, stress relief, and suppression of plant pathogens by induction of plant defenses, production of antibiotics, and/or out-competition of pathogens (Rosenblueth and Martínez-Romero, 2006) (**Figure 3**). To increase field efficiency of microbial inoculation workers have advocated to screen “eco-specific strains” that are acclimatized to a particular set of environmental conditions (Trivedi and Pandey, 2008; Trivedi et al., 2011). This favors efficient establishment and survival of the introduced bacteria leading to increased performance and also does not affect the preexisting balance among indigenous populations.

It has been noticed that certain trees (called escape plants) may survive in heavily infected areas under heavy load of pathogen and vector (Sagaram et al., 2009; Trivedi et al., 2011). Because these escape plants have the same genotype as susceptible plants and have developed under similar edaphic and climatic conditions, a possible explanation for the lack of disease symptoms may lie in the nature of the microbial community associated with these plants. In previous studies, it has been documented that specific endophytic bacterial communities are associated with these escape plants (Sagaram et al., 2009; Bulgari et al., 2011, 2014; Trivedi et al., 2011). Some of the bacteria isolated from these escape plants showed typical traits of potential biocontrol agents (Bulgari et al., 2011; Trivedi et al., 2011). Isolation frequency of bacterial strains showing multiple beneficial activities was higher in escape/healthy as compared to *Ca*. P. mali or Las infected plants (Bulgari et al., 2011; Trivedi et al., 2011). These isolates belonged to *Pseudomonas*, *Bacillus*, and *Lysinibacillus* species and have been previously developed as a carrier based bio-inoculant to increase plant productivity and health of various plant species (Trivedi and Pandey, 2008; Trivedi et al., 2008).

The research on screening effective biocontrol agents against obligate endophytic pathogens such as phytoplasma and *Ca.* Liberibacter spp. is hampered due to the unavailability of proper *in vitro* screening systems that provides repeatable and reliable results in shorter periods of time. The widely used dual culture technique could not be applied to screen bacteria antagonistic to these obligate endophytes due to our inability to culture these bacteria. Trivedi et al. (2009) have developed a method to quantify viable Las with the aid of ethidium mono-azide (EMA) and subsequent qPCR that can differentiate live from dead cells. The EMA-qPCR assay was optimized for screening potential biocontrol bacteria effective against Las (Trivedi et al., 2011). The selected novel isolates are further being tested *in planta* and in field conditions to determine whether they could be used in management of HLB.

Beneficial soil-borne microbes can induce an enhanced defensive capacity in above-ground plant parts to protect plants against insect herbivores (**Figure 3**). This induced systemic resistance (ISR) triggered by soil-borne microbes is often not associated with enhanced biosynthesis of plant hormones that are important for defense against insect herbivores, nor with massive changes in defense-related gene expression. Instead, beneficial soil-borne microbes prime the plants for enhanced defense that is characterized by a faster and stronger expression of defense responses activated upon insect attack, resulting in increased resistance to the insects, and/or decrease in pathogen proliferation (Pieterse et al., 2013). Very recently, the concept of inducing enhanced resistance to phytoplasma with beneficial bacteria has been evaluated using Chrysanthemum as a model organism (Gamalero et al., 2010; D’Amelio et al., 2011; Musetti et al., 2011). Results showed that pretreatment with *Pseudomonas putida* S1Pf1Rif decreases the negative effects on plant growth infected with chrysanthemum yellows phytoplasma (CYP), but had no effect on CYP viability and proliferation (Gamalero et al., 2010). Co-inoculation of *P. putida* S1Pf1Rif and mycorrhizal fungi *Glomus mosseae* BEG12 resulted in a slightly increased resistance and a delay of symptoms in CYP infected and non-resistant plants (D’Amelio et al., 2011). *G. mosseae* could also reduce symptoms of the stolbur phytoplasma causing Bois noir in grapevine and tomato (Lingua et al., 2002). Musetti et al. (2011) demonstrated that the endophyte (*Epicoccum nigrum*) treatment induced ultrastructural changes both in *C. roseus* tissues and in the pathogen and these changes were associated with a lower titer of phytoplasmas in the host plant. Soil-borne microbes can also induce the production of plant hormones such as salicylic acid, which plays a role in plant defense against insect herbivores with a piercing/sucking feeding mode, such as ACP.

Many experiments have demonstrated the growth stimulation of plant crops in the greenhouse, resulting in increased yield parameters and in the control on pathogenic organisms, however, the replication of successful results of PGPMs applications under field conditions has been limited (Antoun and Prévost, 2005; Trivedi and Pandey, 2008; Choudhary and Johri, 2009; Trivedi et al., 2011). The inconsistency in performance (Bashan, 1998; Pandey et al., 1998) may be due to a number of factors but the most important of these are likely to be the differences in the establishment and survival of introduced bacteria (Bashan, 1998; Pandey et al., 1998; Trivedi et al., 2011). Workers have emphasized that understanding ecology, survival, and activity of PGPM’s is a key for their successful field application (Pandey et al., 1998; Trivedi et al., 2005; Trivedi and Pandey, 2008). Formulation of multi-stains of PGPM’s with a broader spectrum of microbial weapons to stimulate plant growth or provide protection against diseases are reported to be more efficient in field conditions compared with single strains (Compant et al., 2005; Gopalakrishnan et al., 2015). Appropriate screening and the application of molecular tools to understand and manage the plant and insect associated microbiome can lead to new products or novel disease management strategies (**Box 1**). One of the key requirements to attain this goal includes a better understanding of interactions between host microbiome and pathogens and to identify key interactions that reduce survival/proliferation of pathogens in host or vectors. This fundamental knowledge can then pave the way to develop new products or tools for sustainable disease management.

**Box 1. Microbiome Engineering to Improve Host Performance and Health.**

Recent breakthroughs in sequencing technologies have provided concrete evidence that the number of microbial cells and the sum of their genetic information are numerically dominant than that of their host. Microbiotas and their hosts interact in a manner that affects the fitness of the holobiont (host genome+microbiome) in many ways, including its morphology, development, physiology, resistance to disease, growth performance, and stress tolerance. Taken together, these interactions characterize the holobiont as a single and unique biological identity. Since that microbiome can adjust more rapidly and by more processes than the host genome to environmental dynamics (including disease progression), it plays fundamental role in the adaptation and fitness of the holobiont (Rosenberg and Zilber-Rosenberg, 2016). Mueller and Sachs (2015) have proposed a novel approach to improve animal and plant fitness by artificially selecting upon microbiomes, thus engineering evolved microbiomes with specific effects on host fitness.

The host-mediated microbiome engineering approach selects upon microbial communities indirectly through the host and leverages host traits that evolved to influence microbiomes (Mueller and Sachs, 2015). Evidence that microbiome can be optimized for disease resistance by the application of phytohormones that activate defense responses is also available (Lebeis et al., 2015). Generating host-mediated artificial selection of microbiomes may be a cheaper way to help curb plant and animal diseases rather than pesticides and antibiotics, or creating genetically modified organisms. Sheth et al. (2016) have highlighted emerging *in situ* genome engineering toolkit to manipulate microbial communities with high specificity and efficacy over a range of specificities and magnitudes. Plant ecological engineering (e.g., integrating plant breeding with microbiome selection) has enormous potential to manipulate host microbiome in order to enhance effectiveness of diseases management.

## Conclusion

The research progress to better understand the interactions between obligate endophytic pathogens belonging to *Ca.* Phytoplasma and *Ca.* Liberibacter species and their hosts and vector has moved slowly because of the inability to isolate these fastidious bacteria on culture media. Studies of plant–pathogen and insect–pathogen interactions are taking advantage from high-throughput techniques and also from the constant improvement of genome sequencing and annotations of both microbes and their hosts (Mitter et al., 2013). However, there is lack of application of these techniques in the area of interaction between host, pathogens and biocontrol agents. Even the most intimate association between the pathogen and its host in the natural environment, whether occurring at the epiphytic or endophytic phase are influenced by a myriad of microbes that are intimately associated with plants or insects. Although it is well documented that various groups of microbes can increase plant productivity in several important crops or defend against pathogen attacks, there are significant challenges that need to overcome in order to harness host associate microbes for sustainable disease management (**Box 2**). Emerging technologies (e.g., next-generation sequencing, new *in vitro* screening tools) combined with well-defined controlled experiments based on evolutionary and ecological theories will facilitate better fundamental understanding on the interaction of pathogens and host associated microbiomes. Furthermore, research on the host-associated microbial community, and its variability, would provide insights into the ecological behavior of pathogenic bacteria in the context of surrounding microorganisms present in the same niches. Such knowledge on multi-trophic microbiome interactions has potential to be harnessed for development of more effective and sustainable management of vector-borne plant diseases.

**Box 2. Priority Challenges.**

Microbiome approach to manage vector mediated plant disease has enormous potential but to achieve this goal, there are some key challenges that need to be solved by integrated fundamental and applied research. These priority challenges include:

(1) Generate improved knowledge of quantitative relationship between endosymbionts of vectors and plants and pathogens
(2) Identify markers which modulate genomic expression of pathogens during switch from host to vectors and vice versa
(3) Discover new tools/chemicals to interrupt signal molecules which facilities pathogen interactions with host and vector.
(4) Develop *in vitro* screening system for biocontrol agents of endophytic pathogens.
(5) Define direction and strength of interaction between plant and vector associated microbiomes and pathogens
(6) Innovate new tools to manipulate vector and host microbiome which can reduce pathogen survival, transfer, and/or proliferation.

## Author Contributions

All authors listed, have made substantial, direct, and intellectual contribution to the work, and approved it for publication.

## Conflict of Interest Statement

The authors declare that the research was conducted in the absence of any commercial or financial relationships that could be construed as a potential conflict of interest.
